# The Roles of Streptozotocin Neurotoxicity and Neutral Endopeptidase in Murine Experimental Diabetic Neuropathy

**DOI:** 10.1155/2009/431980

**Published:** 2010-02-03

**Authors:** Eric Davidson, Lawrence Coppey, Bao Lu, Victor Arballo, Nigel A. Calcutt, Craig Gerard, Mark Yorek

**Affiliations:** ^1^Department of Veterans Affairs Iowa City Health Care System, University of Iowa, Iowa City, IA 52246, USA; ^2^Department of Internal Medicine, University of Iowa, Iowa City, IA 52246, USA; ^3^Ina Sue Perlmutter Laboratory, Department of Pediatrics and Medicine, Harvard Medical School, Children's Hospital, Boston, MA 02115, USA; ^4^Department of Pathology, University of California San Diego, La Jolla, CA 92093, USA

## Abstract

We demonstrated that inhibition of neutral endopeptidase (NEP), a protease that degrades vaso- and neuroactive peptides, improves vascular and neural function in diabetic animal models. In this study we explored the role of NEP in neuropathy related to either insulin-deficient diabetes or diet-induced obesity using NEP deficient (−/−) mice. Initial studies showed that streptozotocin, in the absence of subsequent hyperglycemia, did not induce nerve conduction slowing or paw thermal hypoalgesia. Glucose disposal was impaired in both C57Bl/6 and NEP −/− mice fed a high fat diet. Thermal hypoalgesia and nerve conduction slowing were present in both streptozotocin-diabetic and high fat fed C57Bl/6 mice but not in NEP −/− mice exposed to either streptozotocin-induced diabetes or a high fat diet. These studies suggest that streptozotocin does not induce neurotoxicity in mice and that NEP plays a role in regulating nerve function in insulin-deficient diabetes and diet-induced obesity.

## 1. Introduction

Peripheral neuropathy affects patients with diabetes as well as patients that have impaired glucose tolerance and are considered to be prediabetic [1–5]. Diabetes is the most common cause of peripheral neuropathy [5]. However, about a third of neuropathy patients have no identifiable etiology although 40% of these patients have impaired glucose tolerance [5]. Diabetic neuropathy is considered to be a multifactorial disease arising from interactions between abnormal metabolic activity and impaired vascular function [2, 6, 7]. The etiology of peripheral neuropathy in patients with impaired glucose tolerance is less clear. However, it has been demonstrated that metabolic improvement resulting from aggressive therapy to normalize glucose, hypertension, and hyperlipidemia reduced neuropathic pain in this patient cohort [5].

Our studies have focused on the role of vascular dysfunction in peripheral neuropathy using diabetic animal models as well as animal models of obesity [8–10]. In both type 1 and type 2 diabetic rats we have demonstrated that impaired vascular reactivity precedes the development of nerve dysfunction as identified by reduced nerve conduction velocity [8–11]. Recently we have found that expression of neutral endopeptidase in epineurial arterioles of the sciatic nerve is increased in diabetes and that treating diabetic animal models with AVE7688, a vasopeptidase inhibitor, improves vascular and neural function [12, 13]. Furthermore, it has been demonstrated that vasopeptidase inhibitors are neuroprotective and prevent nephropathy in diabetic rats [14–17]. Vasopeptidase inhibitors are a new class of drug that simultaneously inhibits neutral endopeptidase and angiotensin converting enzyme activity [18]. Neutral endopeptidase degrades a number of vasoactive peptides including natriuretic peptides, adrenomedullin, bradykinin, and calcitonin gene-related peptide [19]. Neutral endopeptidase is found in many tissues including vascular and renal tissues and its activity is increased by fatty acids and glucose in human microvascular cells [20–24]. In the peripheral nervous system neutral endopeptidase is located in Schwann cell membranes surrounding dorsal root ganglion cells and nerve fibers [25, 26]. In the central nervous system neutral endopeptidase is associated with neuronal tissue rather than astrocytes [25]. High levels of the enzyme are present in all neonatal and early postnatal Schwann cells as myelination proceeds it is gradually suppressed in the majority of cells that form myelin but retained in nonmyelin forming cells in the adult animal [26]. Kioussi et al. found that following axonal damage neutral endopeptidase is re-expressed distal to the injury [26]. These authors suggest that neutral endopeptidase could play a role in axonal regeneration [26]. To further explore the role of neutral endopeptidase in peripheral neuropathy induced by diabetes or obesity we performed studies using neutral endopeptidase deficient mice. We hypothesized that because diabetes/obesity would not be able to increase neutral endopeptidase in these mice that nerve function would be protected in diabetic or high fat fed neutral endopeptidase deficient mice. Sciatic nerve conduction velocity slowing and prolonged paw thermal response latency were used as indices of large and small fiber dysfunction, respectively, in both the streptozotocin-treated and high fat fed mouse models of peripheral neuropathy after demonstrating that these disorders were not a consequence of direct streptozotocin-induced neurotoxicity.

## 2. Materials and Methods

Unless stated otherwise all chemicals used in these studies were obtained from Sigma Chemical Co. (St. Louis, MO). 

### 2.1. Animals

 C57Bl/6 wild type mice were purchased from Jackson Laboratories and Swiss Webster mice were purchased from Harlan laboratories. Breeding pairs of neutral endopeptidase deficient (NEP −/−) mice were provided by Lu et al. and are on the C57Bl/6 background [27]. These mice have been bred and a colony created at the Veterans Affairs Medical Center, Iowa City, Iowa. The C57Bl/6 and NEP −/− mice were aged matched for the studies. Deficiency of neutral endopeptidase activity was confirmed in the mice used for these studies by measuring the specific activity of neutral endopeptidase in kidney homogenates using the method described by Ayoub and Melzig [28] with modification. Activity of neutral endopeptidase in kidney from C57Bl/6 and NEP −/− mice was 2.32 ± 0.11 and 0.18 ± 0.06* *μ*M 7-amido-3-methylcoumarin (AMC)/min/*μ*g protein, respectively (*P < .001 versus C57Bl/6 by unpaired *t*-test).

Mice were housed in a certified animal care facility and food (Harlan Teklad, #7001, Madison, WI) and water were provided ad libitum. Adequate measures were taken to minimize pain or discomfort and all of the experiments were conducted in accordance with international standards on animal welfare and were compliant with all institutional and National Research Council's guidelines for use of animals (ACURF protocol 0802033). 

An initial study was performed to address whether streptozotocin causes direct neurotoxicity, independent of inducing insulin deficiency and hyperglycemia. This study was performed by two different laboratories (Yorek and Calcutt) using two different strains of mice. C57Bl/6 and Swiss Webster mice at 12 weeks of age were divided into three groups. The first group was treated with vehicle and served as the control group. The second group was treated with streptozotocin (150 mg/kg i.p. in saline as a single dose to the C57 Bl/6 mice or 90 mg/kg i.p. on two consecutive days to the Swiss Webster mice) to induce diabetes. The third group was treated with 3-O-methylglucose (5.5 mmol/kg body wt, i.p.) approximately one minute prior to each streptozotocin injection to prevent the effects of the streptozotocin on the pancreatic *β* cells [29, 30]. Diabetes was verified 96 hours later by evaluating blood glucose levels with the use of glucose dehydrogenase reagent strips (Accu-Chek, Roche Diagnostics, Indianapolis, IN). Nerve function was assessed after eight (Swiss Webster) or twelve (C57Bl/6) weeks of diabetes. 

For the study examining the influence of deletion of the NEP gene C57Bl/6 and NEP −/− mice at 12 weeks of age were divided into three groups. One group was fed the standard chow diet. A second group was fed a diet containing 60% kcal as fat (D12492, Research Diets, New Brunswick, NJ). The third group was treated with streptozotocin (150 mg/kg i.p. in saline) to induce diabetes. Mice having blood glucose level of 300 mg/dL (16.7 mM) or greater were considered to be diabetic. The experimental period lasted for 12 weeks.

### 2.2. Glucose Tolerance

 Prior to behavioral and nerve conduction studies control and high fat fed mice were fasted overnight for study of glucose utilization. Mice were injected with a saline solution containing 2 g/kg glucose, i.p. Immediately prior to the glucose injection and for 120 minutes afterwards blood samples were taken to measure circulating glucose levels.

### 2.3. Thermal Nociceptive Response

 The day before the terminal studies thermal nociceptive response in the hindpaw was measured using the Hargreaves method with instrumentation provided by IITC Life Science; Woodland Hills, CA (model 390G) or UARD (San Diego). The test was performed when possible in a blind manner. Thermal nociceptive responses were measured by placing the mouse in the observation chamber on top of the thermal testing apparatus and allowing it to acclimate to the warmed glass surface (30°C) and surroundings for a period of 15 minutes. The mobile heat source was maneuvered so that it was under the heal of the hindpaw and then activated, a process that activates a timer and locally warms the glass surface, when the mouse withdrew its paw, the timer, and the heat source was turned off [31]. Following an initial recording, which was discarded, four measurements were made for each hindpaw, with a rest period of 5 minutes between each set of measurements. The mean of the measurements, reported in seconds, was used as a measure of the thermal nociceptive response latency.

### 2.4. Motor and Sensory Nerve Conduction Velocity

 Mice were anesthetized with Nembutal (75 mg/kg, i.p., Abbott Laboratories, North Chicago, IL) or isoflurane (4% in oxygen) and nonfasting blood glucose levels determined with the use of glucose oxidase reagent strips (Lifescan Inc., Milpitas, CA). Afterwards, motor and sensory nerve conduction velocities were determined as previously described [12, 13, 32]. Motor nerve conduction velocity was determined noninvasively in the sciatic-posterior tibial conducting system [12, 13]. Sensory nerve conduction velocity (SNCV) was determined using the digital nerve to the second toe as described by Obrosova et al. [32]. The MNCV and SNCV were reported in meters per second.

### 2.5. Data Analysis

 The results are presented as mean ± SE. Comparisons between the groups for body weight, blood glucose, MNCV, SNCV, and thermal nociception latency were conducted using a one-way ANOVA and Bonferroni's test for multiple comparisons (Prism software; GraphPad, San Diego, CA). A *P*-value of less .05 was considered significant.

## 3. Results

### 3.1. Streptozotocin Neurotoxicity

There has been a considerable amount of discussion about the possible neurotoxic effect of streptozotocin and the suitability of streptozotocin-induced diabetes as an acceptable model for diabetic neuropathy especially in mice when high doses of streptozotocin are used to induce diabetes. To address this issue we used a procedure previously used in rats to protect the pancreatic *β* cells from streptozotocin toxicity [29, 30]. C57Bl/6 and Swiss Webster mice treated with 3-O-methylglucose prior to injection of streptozotocin were normoglycemic and insulin levels were similar to control mice (Table 1). In contrast, mice treated with streptozotocin alone developed hyperglycemia and circulating insulin levels that were significantly decreased. In addition, diabetic mice developed neuropathy, as determined by slowing of nerve conduction velocity and increased response latency to a thermal challenge. Nerve conduction velocity and thermal nociception were normal in 3-O-methylglucose/streptozotocin treated mice. These studies suggest that streptozotocin is not directly neurotoxic in two mouse strains and that nerve function deficits are most likely related to the diabetes-like conditions that develop subsequent to diabetes arising from streptozotocin-induced pancreatic *β* cell damage. 

### 3.2. Role of NEP in Diabetic Neuropathy

Data in Table 2 provide background information on the change in body weight and nonfasting blood glucose level of C57Bl/6 and NEP −/− mice used in this study. The experimental period began when the mice were 12 weeks of age and lasted for 12 weeks. At 12 weeks of age C57Bl/6 mice weighed about 27 g and gained over 4 g during the next 12 weeks. C57Bl/6 mice made diabetic at 12 weeks of age did not gain weight during the experimental period and weighed significantly less and had a greater blood glucose level then control mice at 24 weeks of age. C57Bl/6 mice fed a high fat diet for 12 weeks weighed significantly more then control mice at 24 weeks of age. Neutral endopeptidase deficient mice at 12 weeks of age weighed about the same as C57Bl/6 mice. Like C57Bl/6 control mice, neutral endopeptidase deficient mice gained about 4 g from 12 to 24 weeks of age. The induction of diabetes with streptozotocin in neutral endopeptidase deficient mice was comparable to C57Bl/6 mice. Diabetic neutral endopeptidase deficient mice failed to gain weight and had significantly higher blood glucose levels than control neutral endopeptidase deficient mice. Neutral endopeptidase deficient mice fed a high fat diet for 12 weeks weighed significantly more than control neutral endopeptidase deficient mice at 24 weeks of age and their weight gain was similar to C57Bl/6 mice. Nonfasting blood glucose level was not increased in C57Bl/6 or neutral endopeptidase deficient mice fed a high fat diet.

**Figure 1:**
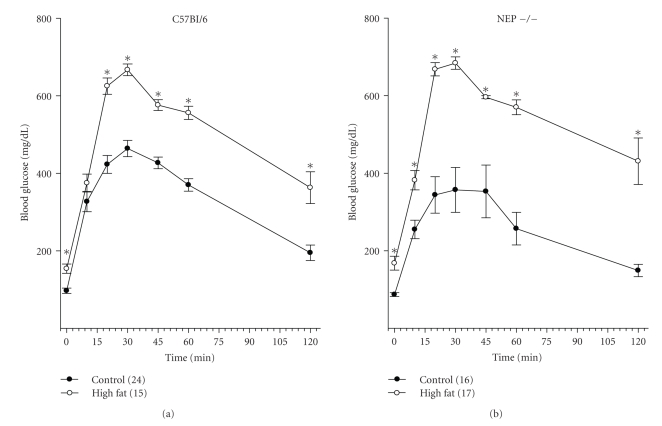
Glucose utilization curve for C57Bl/6 (a) and neutral endopeptidase (NEP −/−) deficient mice (b) fed a normal or high fat containing diet for 12 weeks. Data are the mean ± standard error of the mean. The numbers of animals for each group are indicated in parenthesis. *P < .05 versus control.

Data in Figure 1 demonstrate that C57Bl/6 and neutral endopeptidase deficient mice fed a high fat diet had a significantly higher fasting blood glucose level than respective mice fed a normal diet. Glucose clearance was significantly impaired in C57Bl/6 and neutral endopeptidase deficient mice fed a high fat diet.

**Figure 2:**
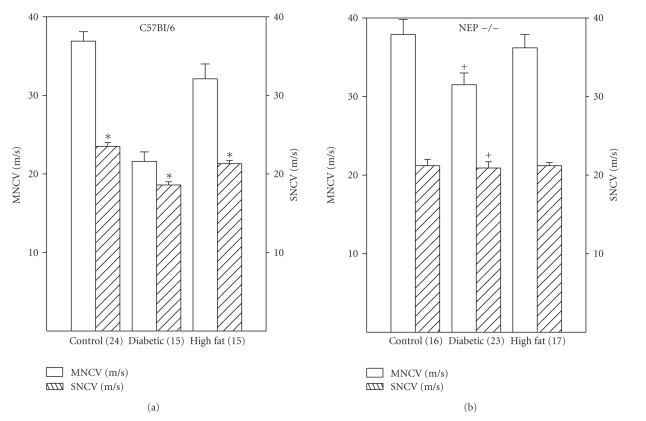
Motor and sensory nerve conduction velocity for C57Bl/6 (a) and neutral endopeptidase (NEP −/−) deficient mice (b). Study groups included mice fed a normal diet for 12 weeks (control), streptozotocin-induced diabetes duration 12 weeks, or fed a high fat containing diet for 12 weeks. Data are the mean ± standard error of the mean. The numbers of animals for each group are indicated in parenthesis. *P < .05 versus control, ^+^ *P* < .05 versus C57Bl/6 mice.

After 12 weeks of untreated diabetes or high fat diet motor and sensory nerve conduction velocity was determined in C57Bl/6 and neutral endopeptidase deficient mice and respective controls. Data in Figure 2 demonstrate that motor and sensory nerve conduction velocity is significantly decreased in diabetic C57Bl/6 mice. In contrast, after 12 weeks of diabetes motor and sensory nerve conduction velocity in neutral endopeptidase deficient mice is not significantly different than control mice and is significantly greater compared to diabetic C57Bl/6 mice. Feeding C57Bl/6 mice but not neutral endopeptidase deficient mice a high fat diet for 12 weeks caused a significant decrease in sensory nerve conduction velocity. Feeding a high fat diet to C57Bl/6 and neutral endopeptidase deficient mice did not change motor nerve conduction velocity.

**Figure 3:**
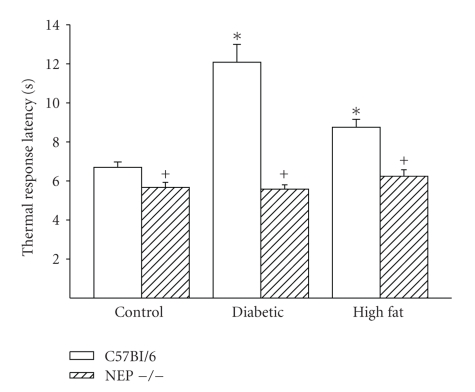
Thermal response latency in the hindpaw for C57Bl/6 and neutral endopeptidase (NEP −/−) deficient mice. Study groups included mice fed a normal diet for 12 weeks (control), streptozotocin-induced diabetes duration 12 weeks, or fed a high fat containing diet for 12 weeks. Data are the mean ± standard error of the mean. The numbers of animals for each group are the same as described in Table 1. *P < .05 versus control C57Bl/6, ^+^ *P* < .05 C57Bl/6 mice versus NEP −/− mice, respectively.

Data in Figure 3 demonstrate that response to a thermal stimulus to the hindpaw is impaired in C57Bl/6 diabetic and high fat fed mice compared to control C57Bl/6 mice. Thus, after 12 weeks of diabetes or high fat diet C57Bl/6 mice are thermal hypoalgesic. In contrast, thermal responsiveness in diabetic or high fat fed neutral endopeptidase deficient mice is not impaired compared to control neutral endopeptidase deficient mice and is significantly more responsive compared to diabetic or high fat fed C57Bl/6 mice, respectively. Furthermore, control neutral endopeptidase deficient mice are more sensitive to a painful stimulus than control C57Bl/6 mice.

## 4. Discussion

Diabetes is the most common cause of peripheral nerve damage rendering both diffuse damage referred to as polyneuropathy and focal damage or mononeuropathy [33, 34]. It is now known that painful sensory neuropathy is also associated with impaired glucose tolerance or metabolic syndrome [35–38]. Animal studies of the pathophysiology of diabetic polyneuropathy have provided us with a long list of mechanisms and possible treatments but these treatments have generally failed in clinical trials [34]. Thus, at this time there is no effective therapy for diabetic polyneuropathy. As described by Zochodne [34], there are likely many reasons for the disappointing results of these clinical trials including choice of agent, trial design, and endpoints. Since the etiology of diabetic polyneuropathy appears to be multifactorial it seems unlikely that a single intervention will be beneficial and a more multitargeted approach is necessary.

My laboratory has focused on the role of microvascular dysfunction in the development and progression of peripheral nerve disorders. Our studies in both type 1 and type 2 diabetic rats and rat models of metabolic syndrome have demonstrated that impaired vascular reactivity precedes the development of nerve dysfunction as identified by reduced nerve conduction velocity [6, 8–11]. Recently we have been examining the role of neutral endopeptidase and the effect of vasopeptidase inhibition in diabetes- and obesity-induced vascular and neural dysfunction. Neutral endopeptidase is found in many tissues including vascular, renal, and neural tissues and its activity is increased by fatty acids and glucose in human microvascular cells [20–26]. Interestingly, neutral endopeptidase activity has been shown to be activated by protein kinase C, which is increased in vascular tissues by diabetes [39, 40]. Neutral endopeptidase degrades vasoactive peptides including natriuretic peptides, adrenomedullin, bradykinin, and calcitonin gene-related peptide [19]. Therefore, use of vasopeptidase inhibitors, a new class of drug that simultaneously inhibits neutral endopeptidase and angiotensin converting enzyme activity, would likely promote expression of vasoactive peptides by blocking degradation and thus, improving vascular function. In this regard, vascular conductance in the femoral artery of streptozotocin-induced diabetic rats was improved by a vasopeptidase inhibitor [41]. Furthermore, it has been demonstrated that vasopeptidase inhibitors are neuroprotective and prevent nephropathy in Zucker diabetic fatty rats [14–17]. Vasopeptidase inhibitors have also been reported to decrease matrix metalloproteinases and AGE accumulation/formation in type 2 diabetes and improve wound healing [42–44]. In our previous studies we have demonstrated that treatment of streptozotocin-induced diabetic rats and Zucker diabetic fatty rats, animal models for types 1 and 2 diabetes, respectively, with AVE7688, a vasopeptidase inhibitor, improved slowing of motor and sensory nerve conduction velocity and vascular impairment [12, 13]. In the previous studies we have also demonstrated that treating streptozotocin-induced diabetic rats or Zucker diabetic fatty rats with Enalapril, an angiotensin converting enzyme inhibitor, improved nerve and vascular dysfunction [45, 46]. However, we concluded that AVE7688 treatment for diabetic neural and vascular complications was more effective than Enalapril [7].

In order to further investigate the role of neutral endopeptidase in peripheral nerve dysfunction we examined the effect of streptozotocin-induced diabetes and diet-induced obesity on nerve conduction velocity and thermal response latency in the hindpaw of C57Bl/6 mice and mice deficient in neutral endopeptidase. The two major findings of these studies are that streptozotocin itself is not neurotoxic and that neutral endopeptidase deficient mice are protected from the slowing of nerve conduction velocity and thermal hypoalgesia that occur in streptozotocin-induced diabetic- or diet-induced obesity-C57Bl/6 mice.

Pabbidi et al. [47] have reported that streptozotocin has a direct action on neurons and modulates the expression and function of TRPV1, a nociceptive ion channel that is responsible for inflammatory thermal pain. In studies performed using dorsal root ganglion neurons they found that hydrogen peroxide mimicked the effects of streptozotocin in regard to increasing the expression of TRPV1. In animal studies they found that some mice injected with streptozotocin (50–200 mg/kg) did not become hyperglycemic but exhibited a transient thermal hyperalgesia using a hot plate test that returned to normal within weeks. This contrasted with hyperglycemic mice that progressed from hyperalgesia to hypoalgesia. In our present studies, mice displayed hypoalgesia 4–12 weeks after injection of streptozotocin and subsequent induction of diabetes. We did not examine our mice earlier than 4 weeks of diabetes, although we have previously reported thermal hypoalgesia as early as 2 weeks after onset of streptozotocin-induced diabetes [48]. Mice treated with 3-O-methylglucose prior to injection of streptozotocin did not demonstrate signs of neuropathy, as indicated by slowing of nerve conduction velocity or altered thermal response latency. We conclude from our studies that streptozotocin alone is not responsible for thermal hypoalgesia or nerve conduction slowing in mice.

Our findings suggest that neutral endopeptidase activity contributes to peripheral neuropathy observed in type 1 diabetes and diet-induced obesity. Slowing of motor and sensory nerve conduction velocity is a common feature in animal models of streptozotocin-induced diabetes [49, 50]. Likewise, it has been demonstrated that C57Bl/6 mice fed a high fat diet for 16 weeks have motor and sensory nerve conduction deficits and thermal hypoalgesia [51].

A reason for the normalcy of nerve conduction velocity in diabetic and obese neutral endopeptidase deficient mice could be the preservation of vascular function. In diabetic and obese rats we have demonstrated that treatment with a vasopeptidase inhibitor prevents vascular dysfunction [12, 13]. This was likely due to protecting vasoactive peptides from degradation by neutral endopeptidase [12, 13, 52, 53]. Preservation of calcitonin gene-related peptide and substance *P* in the dorsal root ganglion of diabetic mice by nerve growth factor has also been shown to prevent/improve diabetic sensory neuropathy [54]. Since neutral endopeptidase degrades both calcitonin gene-related peptide and substance *P* it would be expected that mice deficient in neutral endopeptidase would maintain higher levels of both of these neuroactive peptides and perhaps be protected from neuropathy.

We observed that diabetes- or obesity-induced thermal hypoalgesia of the hindpaw was prevented in neutral endopeptidase deficient mice. In our studies mice were diabetic or fed a high fat containing diet for 12 weeks prior to experimentation. Beiswenger et al. [48] reported that thermal hypoalgesia developed after only 2 weeks of diabetes. Interestingly, they found that a measurable reduction in immunoreactive epidermal nerve fiber density could not be detected until after 4 weeks of diabetes leading them to suggest that impaired epidermal nociceptor function contributes to early diabetes-induced thermal hypoalgesia prior to the loss of peripheral terminals. We propose that increased expression of neutral endopeptidase in epidermal nerve fibers may contribute to epidermal nociceptor dysfunction by increasing degradation of calcitonin gene-related peptide and substance *P* neuroactive peptides involved in pain perception. Neutral endopeptidase is expressed in normal skin and is increased in wounds [55]. In normal skin neutral endopeptidase was localized by immunohistochemistry to keratinocytes of the epidermal basal layer, to hair follicles, eccrine and sebaceous glands, endothelium of blood vessels and large nerves [53]. Furthermore, neutral endopeptidase activity is increased in skin of patients with diabetic ulcers [56]. It is thought that this may contribute to deficient neuroinflammatory signaling and may impair wound healing [56]. We have shown that expression of neutral endopeptidase is increased in epineurial arterioles of the sciatic nerve in diabetic rats [12, 13]. It remains to be determined whether expression/activity of neutral endopeptidase is increased in the epidermis of the footpad of diabetic or obese mice and how this relates to changes in epidermal nerve fiber density.

Normal neutral endopeptidase deficient mice were thermal hyperalgesic compared to control C57Bl/6 mice as determined by latency of withdrawal threshold of the hindpaw. Increased sensitivity to pain stimulus has previously been demonstrated in neutral endopeptidase deficient mice or inhibition of neutral endopeptidase activity [56–58]. It has been shown that neutral endopeptidase knockout in mice induces hyperalgesia induced by bradykinin in a model of visceral pain [57]. Bradykinin is also a peptide degraded by neutral endopeptidase. In studies with human skin inhibition of neutral endopeptidase facilitated neurogenic inflammation and that neutral endopeptidase but not angiotensin converting enzyme was more important for degradation of calcitonin gene-related peptide [58]. Using neutral endopeptidase knockout mice the same group found an increased pain behavior and signs of neurogenic inflammation after soft tissue trauma with and without nerve injury [59]. It is possible that the diabetic or obese neutral endopeptidase deficient mice in our studies did not become thermal hypoalgesic because these mice are more sensitive to a painful stimulus than wild type mice and this sensitivity masked any changes to thermal responsiveness in the hindpaw.

## 5. Conclusions

Using C57Bl/6 and Swiss Webster mice we found that streptozotocin alone is not neurotoxic or responsible for nerve dysfunction associated with streptozotocin-induced diabetes. These studies also provide additional evidence that expression of neutral endopeptidase plays a role in peripheral neuropathy that accompanies diabetes and/or diet-induced obesity. Continued investigation into therapeutic means to manipulate the activity of neutral endopeptidase and perhaps other metalloendopeptidases may lead to an effective treatment for peripheral polyneuropathy.

## Figures and Tables

**Table 1:** Effect of 3-O-methyl-glucose on Markers for Diabetic Neuropathy in Streptozotocin-treated C57Bl/6 and Swiss Webster Mice.

C57Bl/6	Control (10)	Diabetic (15)	3-O-methyl-glucose (15)
Start weight (g)	25. ± 0.7	26.9 ± 0.4	25.4 ± 0.5
End weight (g)	30.6 ± 1.0	25.7 ± 0.8*	29.6 ± 0.5^+^
Blood glucose (mg/dL)	175 ± 8	582 ± 6*	214 ± 27^+^
Insulin (pM)	10.6 ± 3.4	0.7 ± 0.3*	12.8 ± 3.0^+^
MNCV (m/sec)	33.6 ± 2.2	21.6 ± 1.2*	35.7 ± 1.6^+^
SNCV	23.2 ± 0.8	18.6 ±0.4*	22.8 ± 0.5^+^
Thermal response latency (sec)	6.70 ± 0.40	12.08 ± 0.92*	7.21 ± 0.27^+^

Swiss Webster	Control (9)	Diabetic (9)	3-O-methyl-glucose (9)

Start weight (g)	27.8 ± 0.6	29.0 ± 0.7	27.4 ± 0.4
End weight (g)	32.1 ± 1.3	24.6 ± 0.4*	32.3 ± 1.0^+^
Blood glucose (mg/dL)	182 ± 24	573 ± 27*	226 ± 14^+^
MNCV (m/sec)	47.8 ± 1.2	38.8 ± 1.4*	46.4 ± 1.7^+^
Thermal response latency week 4 (sec)	5.6 ± 0.4	8.8 ± 0.7*	5.0 ± 0.6^+^
Thermal response latency week 8 (sec)	4.9 ± 0.4	7.7 ± 0.9*	5.8 ± 0.4

Data are presented as the mean ± SEM. *P < .05 compared to control for the respective group, ^+^ *P* < .05 compared to diabetic. Parentheses indicate the number of experimental animals.

**Table 2:** Weight Change and Blood Glucose Values for C57Bl/6 and NEP −/− Mice.

C57Bl/6	Control (24)	Diabetic (15)	High Fat (15)
Start weight (g)	26.8 ± 0.4	26.9 ± 0.4	28.0 ± 0.4
End weight (g)	31.3 ± 0.5	26.4 ± 0.6*	42.0 ± 1.6*
Blood glucose (mg/dL)	174 ± 4	592 ± 16*	150 ± 4

NEP −/−	Control (16)	Diabetic (23)	High Fat (17)

Start weight (g)	26.7 ± 0.5	26.4 ± 0.6	24.7 ± 0.6
End weight (g)	31.4 ± 0.7	25.0 ± 0.7*	47.6 ± 1.1*
Blood glucose (mg/dL)	169 ± 6	530 ± 25*	184 ± 14

Data are presented as the mean ± SEM. *P < .05 compared to control for the respective group. Parentheses indicate the number of experimental animals.
